# Fatty acid ethyl esters in meconium and language development at 10 and 12 years

**DOI:** 10.1038/s41390-026-04795-x

**Published:** 2026-01-31

**Authors:** Meeyoung O. Min, Barbara A. Lewis, Cynthia F. Bearer, Sonia Minnes, Sun-Kyung Kim, Lynn T. Singer

**Affiliations:** 1 College of Social Work, University of Utah, Salt Lake City, UT, USA; 2 Department of Psychological Sciences, Case Western Reserve University (CWRU), Cleveland, OH, USA.; 3 Department of Pediatrics, UH Rainbow Babies & Children’s Hospital, Cleveland, OH, USA.; 4 School of Medicine, CWRU, Cleveland, OH, USA.; 5 Jack, Joseph and Morton Mandel School of Applied Social Sciences, CWRU, Cleveland, OH, USA.; 6 Department of Population and Quantitative Health Sciences, School of Medicine, CWRU, Cleveland, OH, USA.

## Abstract

**BACKGROUND:**

Prenatal alcohol exposure (PAE) is a major public health concern, yet no reliable clinical tools are available for assessing levels of drinking during pregnancy. Fatty acid ethyl esters (FAEEs), the non-oxidative metabolites of ethanol in meconium, are potential biomarkers for quantifying PAE.

**METHODS:**

In a prospective birth cohort of children exposed to alcohol and drugs in utero, meconium from 216 newborns was analyzed. FAEE concentrations were quantified with gas chromatography via a flame ionization detector. A factor score was derived via a principal component analysis of six FAEE analytes. Expressive and receptive language were assessed in 189 children (56% girls) at ages 10 and/or 12.

**RESULTS:**

Higher FAEE factor scores were related to lower receptive language, with its harmful effect mitigated by non-kinship care at lower levels of FAEEs (*p* = 0.03). This relationship remained significant after adjusting for child IQ (*p* = 0.008). Expressive language showed a non-significant trend (*p* = 0.09), which disappeared after IQ adjustment (*p* > 0.30). Other prenatal drug exposures were unrelated to the effects of FAEEs on language skills.

**CONCLUSIONS:**

Elevated levels of FAEEs in meconium can be potential biomarkers for identifying newborns at risk for poor language development related to PAE.

## INTRODUCTION

Prenatal alcohol exposure (PAE) remains a significant public health problem, as it may negatively affect a developing fetal brain, contributing to lifelong physical and neurological impairments.^1^ Despite no known safe amount of alcohol use during pregnancy, an upward trend of alcohol use among pregnant women has been observed over the last decade in the US,^2^ heightening the concern. Approximately one in seven (13.5%) US pregnant women reported alcohol use within the past month, and 5.2% reported binge drinking (≥4 drinks on a single occasion) in 2018–2020,^3^ an increase from 11.5% on drinking and 3.9% on binge drinking in 2015–2017 and 10.2% and 3.1% in 2011–2013, respectively.^4^

Fetal alcohol spectrum disorders (FASDs) refer to a broad range of physical, neurological, cognitive, behavioral, and developmental disabilities caused by PAE.^5^ The severity of FASD symptoms is related to the level, pattern, and gestational timing of PAE,^5^ with fetal alcohol syndrome (FAS) representing the most severe manifestation of heavy maternal alcohol use during pregnancy. FAS, characterized by postnatal growth restriction, facial dysmorphology, and evidence of central nervous system dysfunction,^6^ is the leading known preventable cause of intellectual disability.^7^ FAS is often recognized later by school personnel.^8^ More prevalent are alcohol-related neurodevelopmental disorders, a subset of FASD, resulting from low-to-moderate levels of drinking during pregnancy, with subtle effects across a wide range of neurocognitive domains, including cognition/intelligence, executive function, language, learning, and self-regulation.^5^ The prevalence estimates for FASD range from 3 to 10% among first graders in the U.S.,^9^ with a much higher estimate of 17% among children in the child welfare system.^10^

Identification of newborns at risk for FASD is critical to facilitate early intervention and minimize secondary disabilities. However, identifying such newborns is challenging, especially when the distinctive facial features of FAS are not present, and without currently reliable clinical tools or consistent diagnostic criteria yet established to determine PAE.^11^ Although structured in-depth interviews may reliably elicit information about the amount, pattern, and gestational timing of PAE,^12^ the nature of self-report is likely to underestimate alcohol use due to selective recall errors, stigma, and social desirability biases,^13^ warranting alternative, objective measures of PAE.

Fatty acid ethyl esters (FAEEs), the non-oxidative metabolites of ethanol analyzed in meconium, have been investigated as biomarkers for identifying alcohol-exposed neonates.^14^ Meconium is an infant’s first stool, and PAE can be detected in meconium as early as 12 weeks of gestation,^14^ as FAEEs accumulate due to ethanol metabolism by the fetus. This non-invasive detection method allows for the identification of moderate and episodic PAE, which is likely to be undetected at birth.

We previously established that higher concentrations of FAEEs in meconium were correlated with maternal alcohol consumption during pregnancy in a low-income urban cohort.^15-17^ Further, in the follow-up of this cohort, higher concentrations of FAEEs in meconium were associated with poorer mental and psychomotor developmental scores on the Bayley Scales of Infant Development at 6.5 months, 1 year, and 2 years of age,^18^ with poorer verbal comprehension, working memory, and Full-Scale IQs on the Wechsler Intelligence Scales for Children at ages 9, 11, and 15 years,^19^ with more caregiver reported aggressive and delinquent behaviors of clinical significance at ages 10 and 12, ^20^ and with a greater likelihood of marijuana use and experiencing substance use problems at age 15,^21^ demonstrating the predictive validity of FAEE as biomarkers of PAE.

Building on our previous findings, the present study aims to explore further validation of FAEEs by examining whether the concentration of FAEEs in infant meconium is related to language development in preadolescence, often observed in studies of PAE in preschoolers^22^ and adolescents.^23^ Language functioning is of particular interest, given the well-established cascading relationships of early language skills to cognitive development^24^ and to subsequent academic and occupational success, social interactions, and adaptive functioning.^25,26^ To date, no studies have examined the association between FAEEs in meconium and language development. Variables that may confound the association between PAE and language development, such as co-occurring prenatal exposure to tobacco, marijuana, and/or cocaine,^27^ psychological distress,^28^ ongoing caregiver substance use, and exposure to violence,^29,30^ were considered in the analysis. Given our prior findings of protective effects of non-kinship foster or adoptive placement early in life against delays in language and cognitive development during the preschool years,^27,31^ childhood,^32,33^ early adolescence^34^ and through emerging adulthood at age 21,^35^ we also explored the association of non-kinship foster or adoptive placement with the outcomes and whether the effects of PAE measured as FAEEs might vary by non-kinship placement. In this cohort of children at high-risk for suboptimal development given maternal drug use, especially cocaine, during pregnancy, non-kinship foster or adoptive care was related to a more enriched caregiving environment with caregivers with higher education levels, lower psychological distress, and lower alcohol and tobacco use than maternal/kinship care.^33,36^ We hypothesized that higher concentrations of FAEEs in meconium would be related to poorer expressive and receptive language development after controlling for relevant confounders.

## METHODS

### Sample and procedure

The present study included 189 children (83 male, 106 female) recruited at birth between September 1994 and June 1996 from a metropolitan teaching hospital for a longitudinal study on the developmental effects of prenatal cocaine exposure.^37,38^ Pregnant women at high risk for drug use, indicated by behavior suggesting intoxication, self-admitted drug use, a lack of prenatal care, or a history of involvement with the Department of Human Services, underwent drug toxicology screenings at delivery per hospital policy. Women with an HIV-positive status, chronic medical illness, a psychiatric history (major depression, bipolar disorder, or schizophrenia), or low intellectual functioning indicated in the medical chart were excluded, as were infants with Down syndrome or congenital heart defects. One infant with FAS was also excluded due to the parent study’s focus on the developmental effects of cocaine. After informed consent, random samples of meconium were obtained from 248 newborns, and 216 had adequate analysis of meconium (≥0.5 g meconium available and ≥50% recovery of internal standard).^17^ Of the 216 children, 14 had missing maternal prenatal substance use interview data, 2 children died by age 10, and 11 dropped out or were lost to contact. The current study utilized data from 189 children who completed language assessments either at age 10 (*n* = 181) or/and at age 12 (*n* = 182), representing 88% retention of the 214 living children, with 174 children completing both assessments. Children and their caregivers were assessed by separate examiners blind to both the mother’s and child’s alcohol and drug exposure status at the developmental research laboratory at 1, 6, and 12 months and 2, 4, 6, 9, 10, 11, and 12 years of age postpartum. A clinical psychologist or master’s level research assistant assessed children, and a social worker or trained research assistant assessed caregivers. Parental written informed consent and child assent were obtained prior to data collection. All participants were compensated with a monetary stipend for their time, lunch, and/or transportation costs. A Certificate of Confidentiality (DA-98-91) was obtained from the Department of Health and Human Services to protect against the release of confidential health information from women participating in the study. The Institutional Review Board of the participating hospital approved the study.

### Measures

#### FAEEs.

Meconium was collected within 24 h after birth and frozen at −70 °C until analysis. FAEEs were extracted with acetone/hexane and isolated using silica gel chromatography. The isolated FAEEs were identified and quantitated by gas chromatography using a flame ionization detector (GC/FID).^15,17^ Six FAEE analytes were examined: ethyl myristate, ethyl palmitate, ethyl oleate, ethyl linoleate, ethyl linolenate, and ethyl arachidonate. Due to skewed distribution, concentrations of each FAEE (ng/g) were transformed by log10 (FAEE + 100). A constant value of 100 was added, so that the value of cases below the limit of detection at 100 could be log-transformed. Given the high correlations between the six FAEE analytes (ranging *r* = 0.83–0.97), we conducted a principal component analysis on the FAEE analytes. One factor was extracted, explaining 92% of the variance. A factor score, a linear combination of the six FAEEs, was calculated for each child. A higher factor score indicates higher concentrations of FAEEs.

#### Prenatal exposure to alcohol, cocaine, tobacco, and marijuana.

At the 1-month postpartum visit, birth mothers were asked to recall the amount and frequency of alcohol and drug use for the month prior to and for each trimester of pregnancy using a Timeline Followback method.^39^ The number of standard drinks (0.5 oz. of absolute alcohol) of beer, wine, or hard liquor per drinking day was computed. The number of drinking days per week was recorded using a Likert-type scale (*0* = not at all to *7* = daily use). The number of drinks per week was calculated by multiplying the number of standard drinks per drinking day by the number of drinking days per week. Risk drinking was assessed via the TWEAK,^40^ with a total score ≥2 indicating risk drinking.^41^ Prenatal cocaine exposure was coded as a dichotomous indicator (yes/no) based on infant meconium or urine, maternal urine, or self-report. It was also quantified based on maternal report of the number of crack cocaine “rocks”consumed and the amount of money spent per day, which was converted to a standard “unit”of cocaine, referring to $20 worth of cocaine. For tobacco and marijuana, the number of tobacco cigarettes smoked per day and marijuana joints smoked per week were collected along with the frequency of use. The alcohol and drug assessment was updated with the child’s current caregiver at each follow-up visit to obtain an assessment of recent (prior 30-day period) postnatal, caregiver alcohol and drug use.

#### Language assessment.

At 10 and 12 years, expressive and receptive language abilities were assessed using the Test of Language Development-Intermediate, 3rd Edition (TOLD-I:3), a standardized assessment designed to evaluate spoken language abilities in children aged 8 years, 0 months to 12 years, 11 months.^42^ TOLD-I:3 is comprised of six subtests: Picture Vocabulary, Malapropisms, Grammatic Comprehension, Generals, Sentence Combining, and Word Ordering. Two composite scores were generated for the current study: Speaking (Expressive Language: Sentence Combining, Word Ordering, and General) and Listening (Receptive Language: Picture Vocabulary, Malapropisms, and Grammatic Comprehension). The composite scores are age-standardized, with a mean of 100 and a standard deviation (SD) of 15.

#### Covariates.

Maternal (age at delivery, race, and education) and child characteristics (sex, race, gestational age, and birth weight, length, and head circumference) were retrieved from hospital birth records and verified at the 1-month postpartum visit. Socioeconomic status (SES) was assessed using the Hollingshead Two-Factor Index^43^ at the 1-month postpartum visit. Maternal psychological distress was assessed using the Global Severity Index (*α* = 0.95), a summary scale of the Brief Symptom Inventory,^44^ at each visit. Maternal receptive vocabulary was assessed using the Peabody Picture Vocabulary Test-Revised (PPVT-R)^45^ at the 1-month postpartum visit and updated using its third edition (PPVT-III)^46^ at later assessments. The child’s placement (with either biological mother/relative or non-kinship adoptive/foster caregiver) was also recorded at each visit, and data on the current caregiver were updated to provide concurrent assessment of caregiver receptive vocabulary, psychological distress, and substance use. At the 9-year visit, children’s intelligence was assessed using the Wechsler Intelligence Scales for Children-Fourth Edition (WISC-IV),^47^ and the quality of the caregiving environment was assessed via caregiver-report of the Home Observation of the Environment.^48^ At the 12-year visit, lifetime frequency of direct and indirect (i.e., witnessing) violence exposure (e.g., beating, robbing, stabbing, or shooting) was assessed using an 8-item questionnaire subscale of the Assessment of Liability and Exposure to Substance Use and Antisocial Behavior, an illustration-based, computer-assisted self-interview for children ages 9–12.^49^ The questions were rated on a six-point scale (*0 = none to 5 = 5 times*), with higher scores indicating greater exposure (*α* = 0.76).

### Statistical analysis

Study variables positively skewed were normalized using a log transformation prior to analyses. Means and SDs were presented by the variables’ original distribution, with transformed data used in analyses. The relationships of the FAEE factor score with language outcomes were evaluated using mixed model repeated measures analyses with maximum likelihood estimation procedures as implemented in SAS Proc Mixed. To account for correlated responses within a subject across time, an unstructured covariance matrix was specified. The unstructured model estimates each variance and covariance uniquely from the data by making no assumptions regarding the nature of the residual correlations between the repeated measures.^50^ Missing data were modeled using full-information maximum likelihood, which utilizes all available information from the observed data. Covariates associated with the FAEE factor score or with the outcomes at *p* ≤ 0.20 for at least one time point were entered into the longitudinal mixed model hierarchically and were retained if, on entry, they were significant at *p* < 0.10 or caused substantial (>10%) change in the FAEE coefficient,^51^ yielding a different set of covariates adjusted on each outcome measure. The FAEE factor score was entered first, followed by other prenatal substance exposures, maternal characteristics (education, psychological distress), child characteristics (sex, race, birth length), and environmental factors (HOME, caregiver PPVT score, non-kinship care, violence exposure). We evaluated the homogeneity of FAEE effects, as well as the effects of non-kinship foster or adoptive placement and other covariates, on children’s language outcomes over time by including an interaction term with time. If the interaction was not significant at *p* < 0.10, the interaction terms were removed from the model.^52^ Given the well-known substantial correlations between general cognitive ability (IQ) and language development, we fitted mixed models without and with IQ to determine whether language skills represent relative deficits that have persisted or are a function of lower cognitive ability. No multicollinearity problems were detected using the variance inflation factor. Two-sided *p* < 0.05 indicates statistical significance.

## RESULTS

### Sample characteristics

The majority of the 189 mothers and children were African American and of low SES (Table 1). Only 12% of mothers were married at the time of the child’s birth. More than one-third (37%, *n* = 70) had not finished high school, with a mean (SD) number of education years of 11.8 (1.5). Of the 189 birth mothers, 107 (57%) reported alcohol use during pregnancy, with 6.9 (12.2) alcohol drinks per week. Of the 107 women who self-reported drinking, 72 (67%) engaged in risk drinking (TWEAK ≥ 2), with 27 mothers reporting ≥7 drinks per week and 15 mothers reporting ≥14 drinks per week. More than half of the mothers (*n* = 115, 61%) smoked cigarettes, 45 (24%) used marijuana, and 86 (46%) used crack cocaine during pregnancy. In terms of birth outcomes (Table 2), the mean (SD) gestational age was 38 (3) weeks with a mean birth weight of 2994 (682) grams. About 10% of the children (*n* = 18) were placed with non-kinship foster or adoptive parents during the first 4 years of life/preschool years; of them, 14 children were all adopted by age 10 and continued to stay in the same non-kinship placement through the 12-year assessments. Among the 14 children in non-kinship adoptive care, 10 children were placed since birth, three children within the first year, and one child at 2 years of age. Among the four children who were placed with non-kinship foster or adoptive care during the first 4 years of life but were placed with biological parents or relatives by age 10, two of them were placed in non-kinship adoptive care at birth until age 9, with two other children placed in non-kinship foster care between ages 4 and 6. The mean (SD) Full-Scale IQ of the study participants was almost 1 SD below the normative mean, with 87 (13) at age 9; the mean (SD) language scores on TOLD:I-3 were more than 1 SD below the normative mean at both 10 and 12 years of age. Table 3 presents bivariate correlations among key variables in the longitudinal model. A higher FAEE factor score was marginally related to a higher maternal GSI score at birth (*r* = 0.13, *p* = 0.08) and to longer birth length (*r* = 0.16, *p* = 0.02). No relationships were found between the FAEE factor score and other prenatal drug exposures (tobacco, marijuana, cocaine; all *p*’s > 0.39).

### Expressive and receptive language at age 10 and 12

Table 4 summarizes the results of the longitudinal analyses on expressive and receptive language abilities at ages 10 and 12 without (Model 1) and with (Model 2) IQ. No interaction terms between the FAEE factor score and age/visits were significant on both expressive and receptive languages, indicating that the association of FAEEs with the language outcomes did not substantially vary across time. Significant decreases were observed in both expressive and receptive language abilities from age 10 to 12 years. The significant likelihood ratio test, χ^2^ (df), indicated that the estimated model provided a significantly better fit than the intercept-only model: χ^2^ (12) = 496, *p* < 0.001 for Model 1 and χ^2^ (13) = 593, *p* < 0.001 for Model 2 on expressive language; χ^2^ (12) = 551, *p* < 0.001 for Model 1 and χ^2^ (13) = 604, *p* < 0.001 for Model 2 on receptive language.

**Fig. 1:**
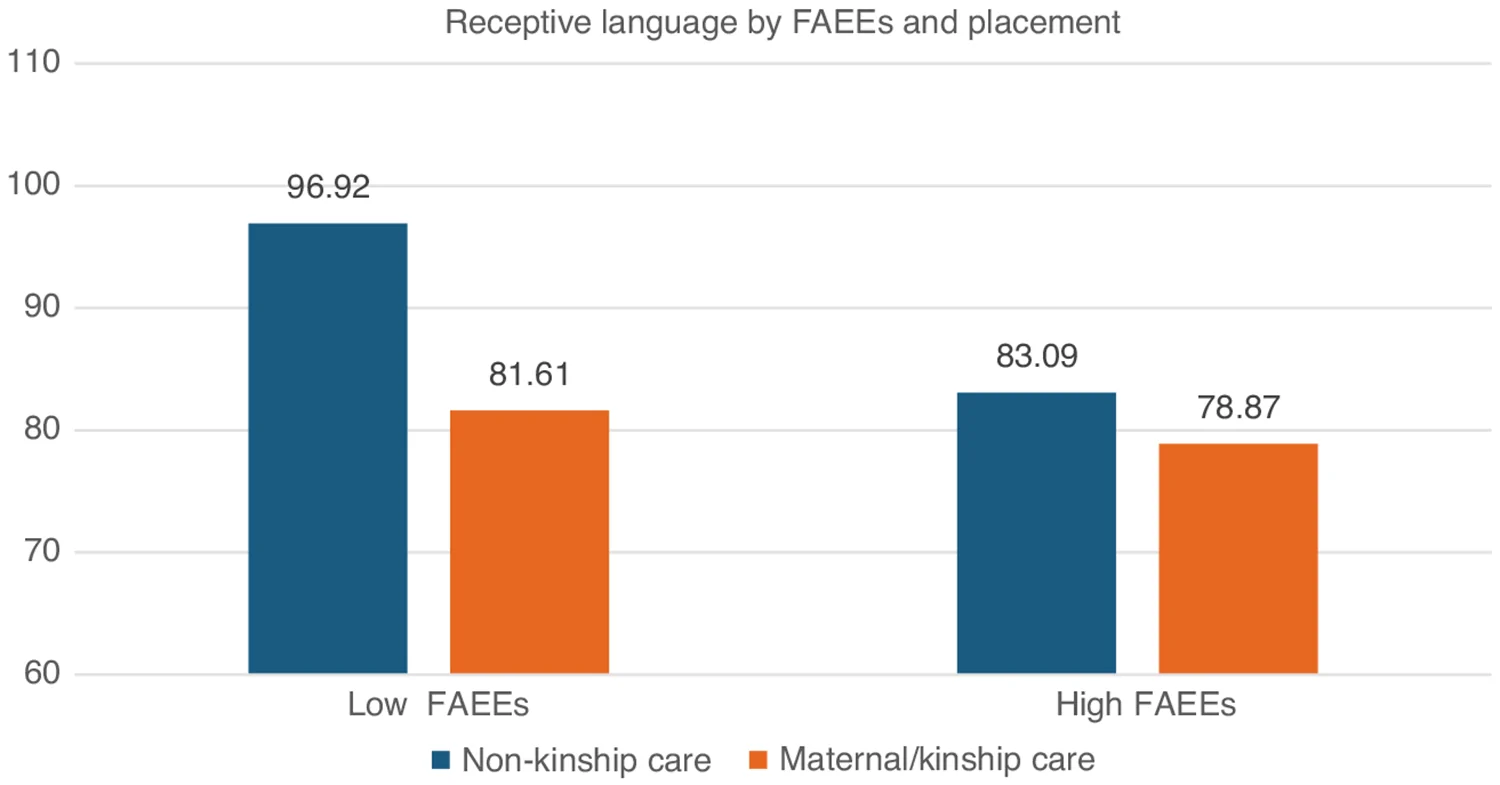
Receptive language by level of FAEEs and placement type at ages 10 and 12. Low FAEEs are defined as FAEE factor scores < median, with high as FAEE factor scores ≥ median. Due to no age-by-FAEEs or age-by-placement interactions, the presented scores represent average receptive language scores over time adjusted for covariates listed in Table 2, Model 1. Significant adjusted mean (*M*_adj_) differences between children in non-kinship care (*n* = 8, *M*_adj_ = 96.92, SE = 4.85) and children in maternal/kinship care (*n* = 84, *M*_adj_ = 81.61, SE = 1.10) in children with low FAEE, *p* = 0.002, and between children with low FAEEs and children with high FAEEs (*n* = 10, *M*_adj_ = 83.09, SE = 3.90) within non-kinship care, *p* = 0.025. No difference between children in non-kinship care and children in maternal/kinship care (*n* = 87, *M*_adj_ = 78.87, SE = 1.15) in children with high FAEE, *p* = 0.30.

A higher FAEE factor score was marginally related to lower expressive language in Model 1, *F* (1,149) = 2.91, *p* = 0.09, but the marginal relationship disappeared when IQ was accounted for in Model 2, *F* (1,150) = 0.75, *p* = 0.39. There was an overall effect of FAEE factor score on receptive language, *F* (1,147) = 8.12, *p* = 0.005, with an interaction between FAEE factor score and non-kinship care during preschool years in Model 1, *F* (1,147) = 4.86, *p* = 0.029. The significance of these relationships did not change when IQ was accounted for in Model 2: the overall effect of the FAEE factor score on receptive language, *F* (1,147) = 8.22, *p* = 0.005, with an interaction between FAEE factor score and non-kinship care during preschool years, *F* (1,147) = 7.35, *p* = 0.008. A higher FAEE factor score was related to lower receptive language, but its harmful effect was mitigated by non-kinship care. Figure 1 illustrates the interaction effect based on Model 1. In children with a FAEE factor score < median (*n* = 92), a significant mean difference in receptive language was found between children in non-kinship care (*n* = 8, *M*_adj_ = 96.92, SE = 4.85) and children in maternal/kinship care (*n* = 84, *M*_adj_ = 81.61, SE = 1.10), *p* = 0.002. However, in children with a high FAEE factor score ≥ median (*n* = 97), the positive impact of non-kinship care disappeared (*p* = 0.30).

In terms of covariates, fewer years of maternal education, lower HOME scores, Black race, and greater exposure to violence were all associated with lower expressive and receptive language in Model 1 without adjusting for IQ. When IQ was adjusted, maternal education and violence exposure were no longer related to expressive and receptive language (Model 2). Longer birth length was related to better receptive language after adjusting for IQ. No other prenatal drug exposures were related to language outcomes.

## DISCUSSION

In this study, higher concentrations of FAEEs in meconium-operationalized as a linear combination of the six FAEE analytes (ethyl myristate, ethyl palmitate, ethyl oleate, ethyl linoleate, ethyl linolenate, and ethyl arachidonate)- were associated with lower receptive language skills in low SES, predominantly Black, urban children who were poly-drug exposed prenatally even after controlling for child IQ. However, non-kinship care early in life mitigated the association of higher levels of FAEEs with poorer receptive language when the concentrations of FAEEs were relatively low, potentially indicating a protective effect of non-kinship care only at lower levels of FAEEs. The more enriched caregiving environment of children in non-kinship care in this sample attenuated the negative relationship of FAEEs to receptive language development at lower FAEE concentration levels. These findings are in alignment with our prior studies, indicating that non-kinship care in this sample was associated with higher HOME scores and caregivers with lower psychological distress and better vocabulary skills than care provided by birth or kinship families.^32-34^ Non-kinship caregivers also used less alcohol and tobacco,^36^ all qualities that likely characterize the protective effect. However, the protective effects of non-kinship care were not apparent at higher levels of PAE as measured through FAEEs. A meta-analysis study of six longitudinal birth cohorts with prospective measures of PAE suggested that an enriched postnatal environment may mitigate the adverse effects of PAE.^53^ The current findings further suggest that the protective effect of a better caregiving environment may be more readily realized at low levels of PAE, a finding that needs to be verified in future studies.

It is puzzling why such a relationship was not observed with expressive language, given that previous studies report deficits in both expressive and receptive languages, especially in early childhood.^22,54^ This discrepancy could be due to (1) different cognitive skills and processes employed by receptive versus expressive language skills^55,56^ and (2) latent “sleeper” effects in PAE, wherein impairment becomes evident later in development as increasing cognitive demands challenge the central nervous system.^57,58^ Receptive language may require more complex integration of auditory processing, working memory- including auditory as well as visual short-term memory- and attentional control,^59,60^ all domains highly vulnerable to PAE-related neurodevelopmental disruption.^53,61^ These cognitive demands of receptive language escalate with age, as children are expected to demonstrate increasingly sophisticated language skills, potentially exceeding their neurocognitive capacity and allowing PAE-associated impairment to become more apparent. A meta-analysis of 12 empirical studies conducted in the US population found that receptive language disorders were more prevalent than expressive language disorders in individuals with FAS.^1^ Furthermore, adolescents aged 12–17 years with heavy PAE (≥4 drinks per occasion at least once per week or >13 drinks per week) scored significantly lower on receptive language compared to peers without PAE, while no differences were observed in expressive language on the Clinical Evaluation of Language Fundamentals-Fifth Edition.^62^ These findings, along with our findings, collectively suggest vulnerability in receptive language to the effects of PAE that may persist into later childhood/adolescence. Future studies examining specific components of receptive and expressive language across developmental stages may capture subtle deficits and clarify the full impact of PAE on language development trajectories.

Mothers in this study who self-reported alcohol consumption were generally moderate to high users, averaging nearly one drink per day, with 67% classified as at-risk drinkers based on TWEAK scores. However, the range of PAE was broad, with the median number of drinks in the low range, and with 40% of the sample reporting no drinking. Our findings align with those of Lewis et al.,^34^ which also observed that PAE was associated with poorer performance on two receptive language subtests of the TOLD- Picture Vocabulary and Grammatical Comprehension- in the present cohort. In that study, PAE was assessed using maternal interview using the Timeline Followback method^63^ at the 1-month postpartum visit.

The present study aimed to examine the direct effects of FAEEs, given evidence from both preclinical and human research demonstrating PAE-related brain alteration,^64,65^ rather than focusing on indirect effects mediated by postpartum environmental factors. Maternal psychological distress and quality of parental care may mediate the impact of FAEE on child language outcomes.^66^ Future research investigating these potential mediating roles could clarify how the effects of PAE may be transmitted through environmental pathways.^67^

We observed that greater violence exposure was related to lower expressive and receptive language skills. Adverse life experiences may alter neuroendocrinological mechanisms, such as the hypothalamic-pituitary-adrenal axis, involved in regulating the stress response, affecting a child’s ability to focus and learn^68^ and interfering with the child’s language acquisition.^29^ Higher quality of home environment and more years of maternal education were also additively related to better expressive and receptive language abilities in children, consistent with numerous studies on language development in children with PAE^22,23^ and without PAE.^69,70^ Collectively, our findings highlight the crucial influence of environmental factors in shaping and modifying language development and even mitigating the deficits caused by PAE.

This study has some limitations. First, for FAEE determination, the use of the GC/FID is less sensitive than tandem mass spectroscopy and may produce false negative detection, or the concentrations measured could be inflated.^14^ Second, since meconium tends to form late in the second and third trimesters of pregnancy, FAEEs in meconium do not capture PAE spanning the entire gestational period, failing to identify newborns of mothers who were able to quit alcohol consumption early in pregnancy. Third, we computed a FAEE factor score to avoid the risk of Type I error due to the high correlations among each FAEE analyte. However, this approach hinders identifying specific FAEE analytes that may be more sensitive to detecting delays in language development. Fourth, given the small number of children placed with non-kinship foster or adoptive parents, this study might lack statistical power for detecting its main effect as well as an interaction effect with FAEE factor scores and be prone to producing Type II error, calling for a replication study with a bigger sample size of children in non-kinship care. Fifth, although biological measures (i.e., meconium and urine) were used to detect prenatal cocaine exposure, other prenatal substance use assessments (tobacco and marijuana) were obtained retrospectively via maternal self-report after delivery and subject to recall error and social desirability bias. Lastly, the lack of data on language interventions that these children may have received might underestimate the influence of FAEEs on the outcomes.

The notable strength of the present study includes its longitudinal prospective birth cohort design, a strong retention rate achieved at preadolescence assessments, use of biological measures to quantify PAE, and a large number of potential confounders evaluated, including other prenatal substance exposures, maternal psychological distress, HOME scores, and violence exposure, all enhancing the credibility of the findings.

In conclusion, elevated levels of FAEEs in meconium can be promising biomarkers for identifying newborns at risk for poor language development related to PAE. Our findings corroborate a convergent body of evidence supporting the validity of FAEEs, which may provide diagnostic clarity for PAE. Future studies should explore thresholds that would predict language delay.

## Figures and Tables

**Table 1.** Biological maternal and caregiver characteristics (*N* = 189)

	*n* (%)/Mean ± SD	Median (10–90%)
*Birth mother*		
Race, Black	152 (80.4)	
Low socioeconomic status	189 (100)	
Years of education	11.80 ± 1.49	12 (10–14)
Married	23 (12.2)	
Age at delivery	27.29 ± 5.30	26 (21–35)
Parity	2.85 ± 1.69	3 (1–5)
Psychological distress	0.67 ± 0.69	0.45 (0.08–1.51)
PPVT Standard Score	76.85 ± 13.28	77 (60–93)
Measures of prenatal alcohol intake (*n* = 107)^a^		
Number of drinks per drinking day	2.26 ± 2.83	1.5 (0.25–5.25)
Number of drinking days per week	1.45 ± 1.50	0.75 (0.12–3.50)
Number of drinks per week	6.91 ± 12.22	2.00 (0.19–20.00)
Risk drinking (TWEAK ≥ 2)	72 (67.3)	
Other prenatal substance use^a^		
Cigarettes per day (*n* = 115)	12.26 ± 10.81 (1.25–20.00)	10.50
Marijuana joints per week (*n* = 45)	2.95 ± 4.31	1.13 (0.13–7.00)
Cocaine units per week (*n* = 86)	24.93 ± 52.09	5.31 (0.38–70.00)
*Caregiver at child age 10*		
HOME, age 9	43.51 ± 6.42	44 (35–51)
Psychological distress	0.34 ± 0.45	0.19 (0.02–0.89)
PPVT Standard Score	80.44 ± 14.00	79 (64–104)
Substance use past 30 days	^a b^	
Alcohol drinks per week (*n* = 59)	2.93 ± 4.22	1.50 (0.25–6)
Cigarettes per day (*n* = 72)	11.13 ± 7.24	10 (3–20)

*PPVT* Peabody Picture Vocabulary Test, *TWEAK* Tolerance, Worried, Eye-openers, Amnesia, and K/Cut down, *HOME* Home Observation for Measurement of the Environment.

a Based on mothers (*n*) who reported the frequency and amount of alcohol intake. Seventy-five mothers reported no drinking during pregnancy, and seven mothers were missing on the frequency and amount of alcohol intake.

b Only two caregivers reported marijuana use, and only one caregiver reported cocaine use in the past 30 days.

**Table 2.** Child characteristics (*N* = 189)

	*n* (%)/Mean ± SD	Median (10–90%)
*At birth*		
Male	83 (43.9)	
Race, Black	152 (80.4)	
Gestational age, weeks	38.35 ± 2.95	39 (35–41)
Prematurity (<37 weeks gestational age)	40 (21.2)	
Birth weight, grams	2994 ± 682	3130 (2000–3735)
Birth length, cm	48.79 ± 3.96	49.0 (44.0–53.0)
Head circumference, cm	33.12 ± 2.41	33.0 (30.2–35.5)
FAEE levels		
Ethyl myristate	551 ± 1708	47 (0–1496)
Ethyl palmitate	949 ± 2335	134 (32–2487)
Ethyl oleate	11,589 ± 36,940	270 (55–33,544)
Ethyl linoleate	17,759 ± 63,645	247 (0–40,614)
Ethyl linolenate	5,147 ± 17,326	129 (0–14,765)
Ethyl archidonate	709 ± 1,409	179 (0–2242)
*At age 10*		
Age at assessment	10.12 ± 0.22	10.1 (10–10.4)
WISC-IV Full-Scale IQ, age 9	86.85 ± 12.94	87 (71–105)
Non-kinship foster/adoptive care by age 4	18 (9.5)	
TOLD-I:3 Language (*n* = 181)		
Expressive language	81.78 ± 14.75	81 (64–102)
Receptive language	84.08 ± 12.40	83 (70–102)
*At age 12*		
Age at assessment	12.09 ± 0.23	12 (12.0–12.4)
Violence exposure^a^	0.63 ± 0.79	0.25 (0–1.88)
TOLD-I:3 Language (*n* = 182)		
Expressive language	78.62 ± 14.07	79 (59–98)
Receptive language	79.37 ± 12.97	79 (64–96)

*WISC-IV* Wechsler Intelligence Scales for Children-Fourth Edition, *TOLD-I:3* The Test of Language Development-Intermediate, 3rd Edition.

a Lifetime frequency, 1 = *none* to 5 = *5 times or more*.

**Table 3.** Pearson correlations among key variables

	Covariates													Language outcomes			
	2	3	4	5	6	7	8	9	10	11	12	13	14	15	16	17	18
1. FAEE factor score	0.03	0.04	0.06	0.13	−0.06	0.04	−0.03	**0.16**	0.06	−0.08	−0.07	0.08	−0.09	−0.09	−0.10	**−0.16**	−0.15
2. Prenatal tobacco^a^	---	**0.27**	**0.52**	**0.23**	**−0.27**	0.05	**−0.32**	**−0.28**	**0.34**	−0.07	**0.18**	0.08	−0.09	−0.09	0.05	−0.04	0.09
3. Prenatal marijuana^a^		---	**0.30**	**0.22**	−0.02	0.08	**−0.15**	**−0.24**	0.06	0.06	0.03	−0.00	−0.03	−0.06	−0.01	−0.12	0.03
4. Prenatal cocaine, yes			---	**25**	**−0.18**	0.00	0.08	**−0.27**	**0.25**	−0.08	−0.12	0.06	−0.11	−0.14	−0.10	−0.10	−0.06
5. Maternal psychological distress^a^				---	**−0.20**	−0.05	−0.14	−0.09	**0.21**	−0.06	−0.10	−0.06	−0.03	−0.08	−0.09	−0.12	−0.07
6. Maternal education					---	0.03	**0.24**	0.02	**−0.25**	**0.16**	0.10	−0.10	**0.22**	**0.15**	**0.17**	**0.17**	**0.16**
7. Child sex, male						---	−0.13	0.02	0.08	**−0.16**	0.14	0.08	−0.05	−0.10	0.04	−0.05	0.08
8. Child race, Black							---	**−0.15**	**−0.16**	−0.00	**−0.33**	0.14	**−0.20**	**−0.24**	**−0.34**	**−0.24**	**−0.40**
9. Birth length								---	**−0.22**	−0.02	0.06	0.07	**0.23**	**0.20**	0.14	0.14	0.11
10. Non-kinship care by age 4									---	0.10	**0.29**	−0.05	−0.02	0.12	**0.20**	0.08	**0.21**
11. HOME										---	**0.17**	−0.09	**0.22**	**0.25**	**0.35**	**0.25**	**0.22**
12. Caregiver PPVT, age 10											---	−0.11	0.12	**0.23**	**0.33**	**0.25**	**0.29**
13. Violence exposure												---	**−0.22**	**−0.15**	**−0.22**	**−0.25**	**−0.30**
14. IQ													---	**0.73**	**0.61**	**0.73**	**0.61**
15. Expressive language, age 10														---	**0.76**	**0.84**	**0.70**
16, Receptive language, age 10															---	**0.74**	**0.82**
17. Expressive language, age 12																---	**0.77**
18. Receptive language, age 12																	---

Bold indicates significance at *p* < 0.05.

*GSI* Global Severity Index, *HOME* Home Observation for Measurement of the Environment, *PPVT* Peabody Picture Vocabulary Test.

a Log-transformed due to skewness.

**Table 4.** Association of FAEEs with expressive and receptive language at age 10 and 12 years

	Expressive language						Receptive language					
	Model 1			Model 2			Model 1		Model 2			
	*b*	SE	*p*	*b*	SE	*p*	*b*	SE	*p*	*b*	SE	*p*
Intercept	35.08	17.56	.048	7.91	13.06	0.55	30.80	14.99	0.029	15.64	11.83	0.19
FAEE factor score	−1.77	1.04	0.090	−0.67	0.77	0.39	−6.86	2.57	0.009	−6.30	2.14	0.004
Prenatal marijuana exposure	−3.05	1.77	0.087	−1.25	1.30	0.34	-	-	-	-	-	-
Prenatal cocaine exposure, yes	−0.72	2.12	0.73	−0.63	1.55	0.68	−0.48	1.64	0.77	0.19	1.37	0.89
Maternal psychological distress	−4.14	2.96	0.16	−3.97	2.17	0.07	−4.03	2.33	0.086	−3.90	1.94	0.046
Maternal education	1.64	0.69	0.019	0.11	0.53	0.83	1.44	0.56	0.011	0.51	0.49	0.29
Time, age 10	3.31	0.65	<0.001	3.33	0.64	<0.001	4.96	0.64	<0.001	4.96	0.63	<0.001
Child sex, male	−1.47	1.97	0.46	−1.49	1.45	0.30	-	-	-	-	-	-
Child race, Black	−11.97	2.69	<0.001	−5.68	2.05	0.006	−13.06	2.18	<0.001	−9.32	1.87	<0.001
Birth length	0.33	0.29	0.25	0.09	0.21	0.68	0.61	0.23	0.008	0.38	0.19	0.047
HOME	0.50	0.15	0.001	0.22	0.11	0.053	0.54	0.12	<0.001	0.37	0.10	<0.001
Caregiver PPVT score	-	-	-	-	-	-	0.02	0.06	0.72	0.02	0.05	0.68
Non-kinship care by age 4	5.63	3.94	0.15	4.79	2.89	0.10	10.66	3.36	0.002	9.70	2.81	<0.001
FAEE*Non-kinship care	-	-	-	-	-	-	−5.88	2.67	0.029	−6.02	2.22	0.008
Violence exposure	−2.51	1.22	0.041	−0.45	0.90	0.62	−2.07	0.96	0.033	−0.99	0.81	0.22
IQ	-	-	-	0.72	0.06	<0.001	-	-	-	0.46	0.06	<0.001

Blank spaces indicate that the variable did not meet the criteria (e.g., not significant at the bivariate level) and therefore was not included in the model.

*HOME* Home Observation for Measurement of the Environment, *PPVT* Peabody Picture Vocabulary Test.

## Data Availability

The datasets generated during and/or analyzed during the current study are not publicly available due to privacy or ethical restrictions, but are available from the corresponding author on reasonable request.

## IMPACT:

- Higher concentrations of Fatty acid ethyl esters (FAEEs), the non-oxidative metabolites of ethanol, analyzed in meconium, are associated with lower receptive language at 10 and 12 years of age, but their harmful effect was mitigated at lower levels of FAEEs by the more enriched caregiving environment of non-kinship care.
- Elevated levels of FAEEs in meconium can be potential biomarkers for identifying newborns at risk for poor language development related to prenatal alcohol exposure (PAE).
- FAEEs may provide diagnostic clarity for PAE, allowing the identification of moderate and episodic PAE, which is unlikely to be detected at birth.
